# Antidepressants and antibiotic resistance in urine cultures: a cohort study

**DOI:** 10.1017/ash.2025.73

**Published:** 2025-04-23

**Authors:** Isaac Sears, Michel Davis, Kevin Gibas, Leonard Mermel, Daithi S. Heffernan

**Affiliations:** 1 The Warren Alpert Medical School of Brown University, Providence, RI, USA; 2 Department of Medicine, The Warren Alpert Medical School of Brown University, Providence, RI, USA; 3 Department of Epidemiology and Infection Prevention, Rhode Island Hospital, Providence, RI, USA; 4 Department of Surgery, The Warren Alpert Medical School of Brown University, Providence, RI, USA

## Abstract

*In vitro* evidence of antidepressant-driven antibiotic resistance has recently been described. In this retrospective cohort study, significant associations are identified between antidepressant use and antibiotic resistance on urine cultures taken in the Emergency Department. This epidemiologic data supports previous *in vitro* work and raises additional questions for further study.

## Introduction

Approximately 2.8 million antibiotic-resistant infections are diagnosed in the US yearly, resulting in approximately 35,000 deaths and $20 billion in medical costs.^1^ Additionally, approximately 60 million antidepressant prescriptions are filled each year.^2^ The impact of antidepressant use on the development of antibiotic resistance has recently been described. *Escherichia coli* exposed to antidepressants promoted antibiotic resistance^3^ by accelerating horizontal transfer of antibiotic resistance genes^4^ and stimulating efflux pump activity.^5^ To establish a clinical association between antidepressant use and antibiotic resistance, we assessed whether a significant increase in antibiotic resistance would be observed in patients taking antidepressants.

## Methods

This is a retrospective analysis of the emergency department (ED) module of the Medical Information Mart for Intensive Care version IV (MIMIC-IV) database, a publicly available database with de-identified medical record data pertaining to presentations at Beth Israel Deaconess Medical Center from 2011 through 2019.^6^ ED encounters in which urine cultures were taken, and grew *E. coli* were selected for the study. For patients who presented with multiple encounters that fulfilled the inclusion criteria, only the first was used so as not to violate assumptions of independence necessary for robust logistic regression analysis. ED encounters in which antibiotics were administered before urine cultures were collected were excluded. Antidepressant use was determined by extracting medication reconciliation records for each encounter. These records were queried for the presence of the 10 most prescribed antidepressants in the database: amitriptyline, bupropion, citalopram, duloxetine, escitalopram, fluoxetine, mirtazapine, sertraline, trazodone, and venlafaxine. Antibiotic resistance was determined by extracting sensitivity data from the urine culture results for 3 antibiotics commonly used to treat urinary tract infections: fluoroquinolones (any), trimethoprim-sulfamethoxazole, and nitrofurantoin. A series of 3 logistic regression models assessed the association between antidepressants and the presence of resistance to each of the specific antibiotics. Models controlled for the presence of available potential confounders: age, sex, concurrent use of any antibiotic, hospitalization in the past year (BIDMC only), Charlson comorbidity index, and pyelonephritis.

## Results

**Figure 1.**
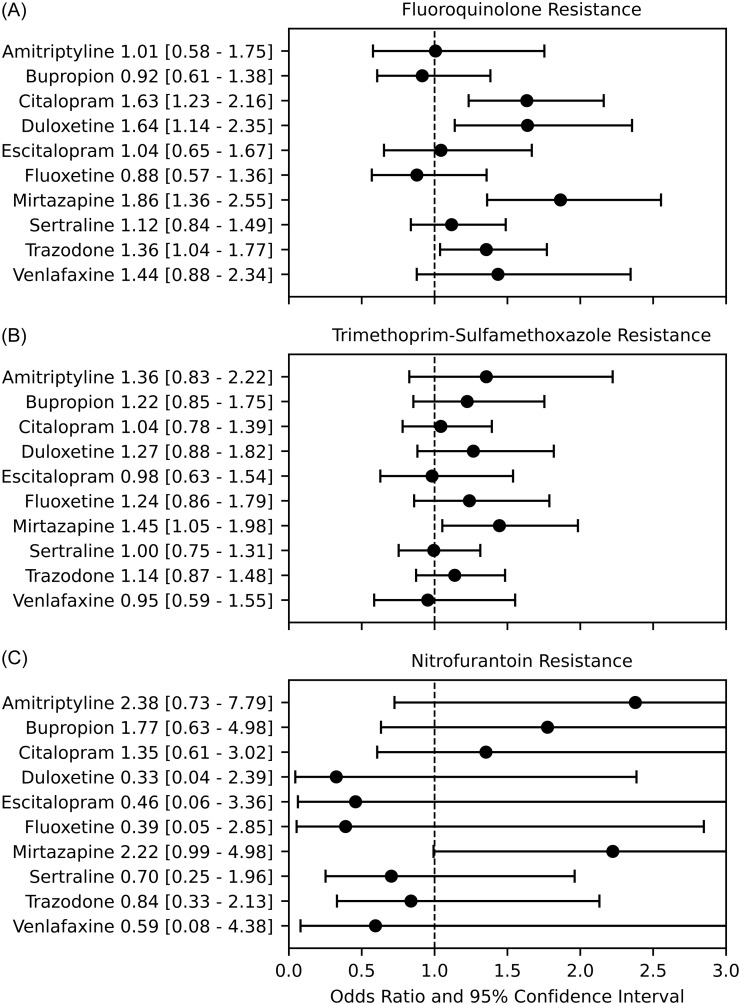
Adjusted odds of resistance to Fluoroquinolones (A), Trimethoprim-Sulfamethoxazole (B), and Nitrofurantoin (C) by antidepressant.

5,157 ED encounters were identified with urine cultures positive for *E. coli* (Table 1). The mean age of included subjects was 62 (+/– 22) years, and 4,191 (81%) were female. 1,370 (27%) encounters involved patients who were taking antidepressants. 1,204 (23%) cultures demonstrated fluoroquinolone resistance, 1,402 (27%) demonstrated trimethoprim-sulfamethoxazole resistance, and 88 (2%) demonstrated nitrofurantoin resistance. Among patients not taking antidepressants, the figures were 776 (20%), 986 (26%), and 60 (2%), respectively. Four antidepressants were associated with significantly increased adjusted odds of fluoroquinolone resistance: citalopram (OR 1.63 [95% CI, 1.23–2.16]), duloxetine (1.64 [95% CI, 1.14–2.35]), mirtazapine (OR 1.86 [95% CI, 1.36–2.55]), and trazodone (OR 1.36 [95% CI, 1.04–1.77]) (Figure 1). Only mirtazapine was associated with trimethoprim-sulfamethoxazole resistance (OR 1.45 [95% CI, 1.05–1.98]). There were no significant associations for nitrofurantoin resistance. No antidepressant was associated with decreased odds of antibiotic resistance.

**Table 1.** Subject characteristics

	Fluoroquinolone resistance (n=1,204)	Trimethoprim-sulfamethoxazole resistance (n=1,402)	Nitrofurantoin resistance (n=88)	No resistance (n=3,165)	All (n=5,157)
**Demographics**					
Age, mean (SD), yr	69 (18)	62 (22)	67 (20)	61 (23)	62 (22)
Female	893 (74)	1090 (78)	67 (76)	2633 (83)	4191 (81)
Male	311 (26)	312 (22)	21 (24)	532 (17)	966 (19)
**Clinical characteristics**					
Concurrent antibiotic use, no. (%)	113 (9)	101 (7)	12 (14)	134 (4)	287 (6)
Hospitalization at Beth Israel Deaconess Medical Center in the past year, no. (%)	248 (21)	241 (17)	10 (11)	513 (16)	861 (17)
Diagnosis of pyelonephritis no. (%)	36 (3)	85 (6)	3 (3)	229 (7)	329 (6)
Charlson comorbidity index, mean (SD)	3 (2)	2 (2)	3 (2)	2 (2)	2 (2)
**Antidepressant use no. (%)**					
Amitriptyline	18 (1)	25 (2)	3 (3)	38 (1)	72 (1)
Bupropion	33 (3)	47 (3)	4 (5)	81 (3)	143 (3)
Citalopram	91 (8)	71 (5)	7 (8)	130 (4)	248 (5)
Duloxetine	52 (4)	47 (3)	1 (1)	76 (2)	143 (3)
Escitalopram	27 (2)	28 (2)	1 (1)	61 (2)	100 (2)
Fluoxetine	29 (2)	45 (3)	1 (1)	85 (3)	141 (3)
Mirtazapine	79 (7)	66 (5)	7 (8)	80 (3)	181 (4)
Sertraline	75 (6)	76 (5)	4 (5)	160 (5)	273 (5)
Trazodone	100 (8)	91 (6)	5 (6)	150 (5)	288 (6)
Venlafaxine	25 (2)	23 (2)	1 (1)	50 (2)	86 (2)
Any	428 (36)	416 (30)	28 (32)	755 (24)	1,370 (27)
No antidepressant	776 (64)	986 (70)	60 (68)	2,410 (76)	3,787 (73)
**Antibiotic resistance and co-resistance no. (%)**					
Fluoroquinolone	1,204 (100)	643 (46)	51 (58)	0 (0)	1,204 (23)
Trimethoprim-sulfamethoxazole	643 (53)	1,402 (100)	37 (42)	0 (0)	1,402 (27)
Nitrofurantoin	51 (4)	37 (3)	88 (100)	0 (0)	88 (2)

## Discussion

This study demonstrates an epidemiologic association between antibiotic resistance in *E. coli* isolates from urine cultures and the use of commonly prescribed antidepressants. Prior in vitro work assessing the relationship between antidepressants bupropion, escitalopram, duloxetine, and sertraline and *E. coli* resistance found a particularly strong association between duloxetine exposure and fluoroquinolone resistance.^3^ This evidence parallels our findings that duloxetine is associated with fluoroquinolone resistance but not trimethoprim-sulfamethoxazole resistance. But, in contradistinction to the in vitro data, we found no epidemiologic association between sertraline use and antibiotic resistance. Our epidemiologic data may be more reflective of the properties of antidepressant metabolites as they appear in urine than the unmodified antidepressants studied in vitro. Both duloxetine and sertraline undergo extensive hepatic metabolism through different pathways before urinary excretion, with minimal amounts of unchanged drug in the urine.^7,8^

Our findings suggest that mirtazapine has the broadest association with antibiotic resistance, especially fluoroquinolone and trimethoprim-sulfamethoxazole resistance. To our knowledge, no study has investigated the association between mirtazapine and the development of antibiotic resistance in vitro. There is some evidence that mirtazapine inhibits *E. coli* growth in vitro, but not significantly greater than venlafaxine.^9^ This discrepancy may also be attributable to the differing properties of mirtazapine and its urine metabolites but additionally opens the possibility of a new mechanism of antibacterial resistance stimulation unique to mirtazapine.

This study has limitations. Data about indications, dosages, and durations of outpatient antidepressants or antibiotics were not available in the MIMIC database to stratify the analysis by those variables. In addition, 20% of patients were not represented in the medication reconciliation report, and it is unclear if they were not taking any home medications or if the information was not collected. We opted to include these patients in the study so as not to bias the study towards the population taking outpatient medications, but in doing so, we may be including some patients for whom we have incomplete information. Lastly, as a single-center study does not reflect regional resistance patterns in other institutions and the associations presented may be impacted by unmeasured confounders not available in this dataset.

Urinary tract infections are often treated empirically based on local drug resistance patterns.^10^ While not strong enough to alter current clinical recommendations, this work lays the foundation for future guideline considerations. In vitro studies that investigate antidepressant urine metabolites may correlate better with epidemiologic findings. There is also a lack of in vitro data on the association between mirtazapine and antibiotic resistance development. In the context of increasing rates of antibiotic-resistant infections, as well as growing antidepressant use, this phenomenon merits further study.

## Supporting information

Sears et al. supplementary material Sears et al. supplementary material

## Supplementary material

For supplementary material accompanying this paper visit http://doi.org/10.1017/ash.2025.73. click here to view supplementary material
