# Supplementary material for: Antidepressants and antibiotic resistance in urine cultures: a cohort study
Source: Antimicrob Steward Healthc Epidemiol. 2025 Apr 23;5(1):e98. doi: 10.1017/ash.2025.73 (PMC12022929; doi:10.1017/ash.2025.73)
Supplement: Sears et al. supplementary material [file S2732494X25000737sup001.docx]

**Supplemental Table 1.** Adjusted and Unadjusted Odds of Resistance

|  | **Unadjusted Odds of Resistance (CI)** | **Adjusted Odds of Resistance (CI)** |
| --- | --- | --- |
| **Fluoroquinolone Resistance** | | |
| Amitriptyline | 1.10 [0.60 - 1.91] | 1.01 [0.58 - 1.75] |
| Bupropion | 0.98 [0.64 - 1.47] | 0.92 [0.61 - 1.38] |
| Citalopram | 1.98 [1.50 - 2.60] | 1.63 [1.23 - 2.16] |
| Duloxetine | 1.92 [1.33 - 2.74] | 1.64 [1.14 - 2.35] |
| Escitalopram | 1.22 [0.75 - 1.93] | 1.04 [0.65 - 1.67] |
| Fluoxetine | 0.85 [0.54 - 1.29] | 0.88 [0.57 - 1.36] |
| Mirtazapine | 2.65 [1.94 - 3.62] | 1.86 [1.36 - 2.55] |
| Sertraline | 1.26 [0.94 - 1.67] | 1.12 [0.84 - 1.49] |
| Trazodone | 1.81 [1.40 - 2.35] | 1.36 [1.04 - 1.77] |
| Venlafaxine | 1.35 [0.81 - 2.20] | 1.44 [0.88 - 2.34] |
| **Trimethoprim-Sulfamethoxazole Resistance** | | |
| Amitriptyline | 1.43 [0.84 - 2.38] | 1.36 [0.83 - 2.22] |
| Bupropion | 1.32 [0.91 - 1.90] | 1.22 [0.85 - 1.75] |
| Citalopram | 1.08 [0.80 - 1.44] | 1.04 [0.78 - 1.39] |
| Duloxetine | 1.32 [0.91 - 1.90] | 1.27 [0.88 - 1.82] |
| Escitalopram | 1.04 [0.65 - 1.64] | 0.98 [0.63 - 1.54] |
| Fluoxetine | 1.28 [0.87 - 1.85] | 1.24 [0.86 - 1.79] |
| Mirtazapine | 1.56 [1.13 - 2.15] | 1.45 [1.05 - 1.98] |
| Sertraline | 1.03 [0.78 - 1.37] | 1.00 [0.75 - 1.31] |
| Trazodone | 1.25 [0.96 - 1.63] | 1.14 [0.87 - 1.48] |
| Venlafaxine | 0.98 [0.58 - 1.60] | 0.95 [0.59 - 1.55] |
| **Nitrofurantoin Resistance** | | |
| Amitriptyline | 2.56 [0.50 - 8.04] | 2.38 [0.73 - 7.79] |
| Bupropion | 1.69 [0.44 - 4.58] | 1.77 [0.63 - 4.98] |
| Citalopram | 1.73 [0.67 - 3.79] | 1.35 [0.61 - 3.02] |
| Duloxetine | 0.40 [0.01 - 2.32] | 0.33 [0.04 - 2.39] |
| Escitalopram | 0.58 [0.01 - 3.38] | 0.46 [0.06 - 3.36] |
| Fluoxetine | 0.40 [0.01 - 2.35] | 0.39 [0.05 - 2.85] |
| Mirtazapine | 2.43 [0.93 - 5.35] | 2.22 [0.99 - 4.98] |
| Sertraline | 0.85 [0.22 - 2.28] | 0.70 [0.25 - 1.96] |
| Trazodone | 1.02 [0.32 - 2.50] | 0.84 [0.33 - 2.13] |
| Venlafaxine | 0.67 [0.02 - 3.96] | 0.59 [0.08 - 4.38] |
